# People Gather Only the Information They Need to Make Decisions

**DOI:** 10.1371/journal.pbio.1001204

**Published:** 2011-11-22

**Authors:** Janelle Weaver

**Affiliations:** Freelance Science Writer, Glenwood Springs, Colorado, United States of America; PLoS, United States of America

**Figure pbio-1001204-g001:**
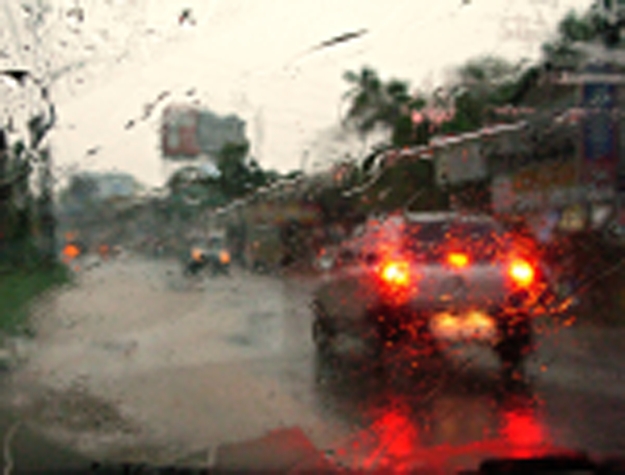
The study by de Lange et al. examines how decision-making is altered by object visibility. People sometimes find this process difficult, for example, when they can barely perceive road signs under poor conditions.

[Fig pbio-1001204-g001]Imagine that you're driving down the freeway on a foggy day. Struggling to read an exit sign, you stare at it for several seconds before you can make out enough of the letters to determine that it's the right exit. In the past, you hardly glanced at the same sign when using that off-ramp.

This phenomenon illustrates an adaptive feature of brain function. To conserve energy, you should exert a minimal amount of effort extracting information from your visual surroundings to make a decision. In general, objects require more processing when they are obscured than when they are clear.

Behavioral and neural evidence for this principle is provided in a paper published this month in *PLoS Biology*. A team led by Floris de Lange, a cognitive neuroscientist at Radboud University Nijmegen in The Netherlands, demonstrated that the degree of object visibility determines the extent to which people absorb incoming information and use it to make decisions. The results suggest that the brain uses more flexible and sophisticated strategies of accumulating evidence than previously appreciated.

In a given trial within the study, volunteers saw on a screen in front of them a series of five briefly flashed arrows that each pointed either left or right. Their task was to indicate the prevailing direction of the five arrows by pressing a button as quickly as possible. The arrows were easy to see in half of the trials, but in the other half, another image that appeared shortly after the arrows reduced their visibility.

For both the high and low visibility trials, accuracy was better when more arrows in a set pointed in the same direction, suggesting that individuals acquired evidence from the sequential images to make a decision. But they processed information more efficiently when the arrows were easy to see. In the high visibility condition, reaction times were faster in trials with more arrows pointing in the same direction, or a greater amount of accumulated evidence.

Additional benefits were observed in the high visibility condition. In each trial, the impact of the last arrow on the decision was lower when the subjects amassed more evidence, as if they disregarded new input once they obtained sufficient information. By contrast, the barely perceptible arrows contributed equally to a decision because the subjects needed to glean as much information as possible during each of these challenging trials.

These findings are supported by data from a noninvasive recording technique called magnetoencephalography, which measures magnetic fields generated by neuronal activity. Once the subjects acquired sufficient evidence in a trial, subsequent images evoked less activity in visual regions of the brain, but only in the condition with highly visible arrows. Moreover, these stimuli produced enhanced signals that originated from brain regions in the frontal lobe and spread to visual regions in the occipital lobe. The frontal regions, which are involved in decision-making and other complex tasks, may essentially instruct the visual regions to ignore additional input once enough information accrues.

These results suggest that people deploy rational decision-making strategies by adapting how much new input they assimilate, depending on the quality and quantity of past evidence. This concept could explain why the exact same object, such as a freeway exit sign, can require more or less processing by the nervous system under different circumstances.

**de Lange FP, van Gaal S, Lamme VAF, Dehaene S (2011) How Awareness Changes the Relative Weights of Evidence during Human Decision-Making. doi:10.1371/journal. pbio.1001203**

